# Supplementary material for: Fluorescence intermittency originates from reclustering in two-dimensional organic semiconductors
Source: Nat Commun. 2017 Feb 22;8:14521. doi: 10.1038/ncomms14521 (PMC5322502; doi:10.1038/ncomms14521)
Supplement: Supplementary Information — Supplementary Figures, Supplementary Tables, Supplementary Notes, Supplementary Methods and Supplementary References [file ncomms14521-s1.pdf]

Supplementary Information

Supplementary Figures

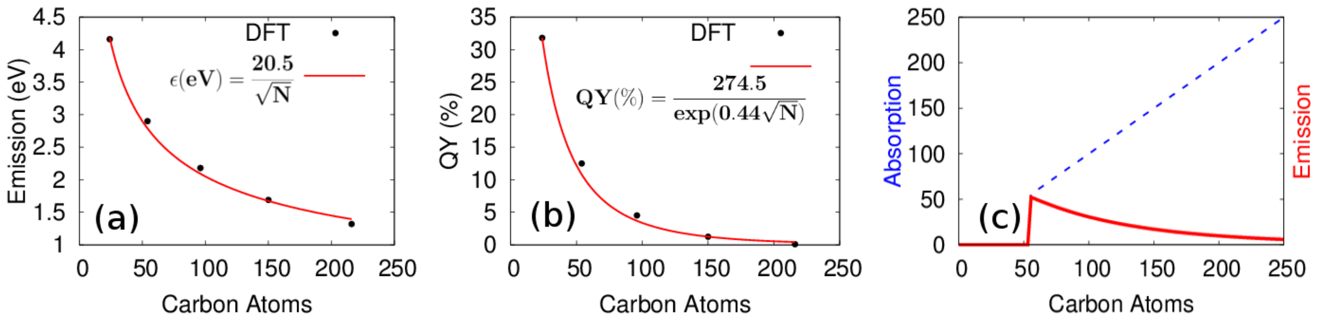

Supplementary Figure 1. Calibration curves from DFT results for Monte Carlo simulations The black dots labeled DFT come from Supplementary Table 1. (a) Emission energy vs. cluster size (b) Quantum Yield vs. cluster size (c) Emission intensity and absorption cross section vs. cluster size.

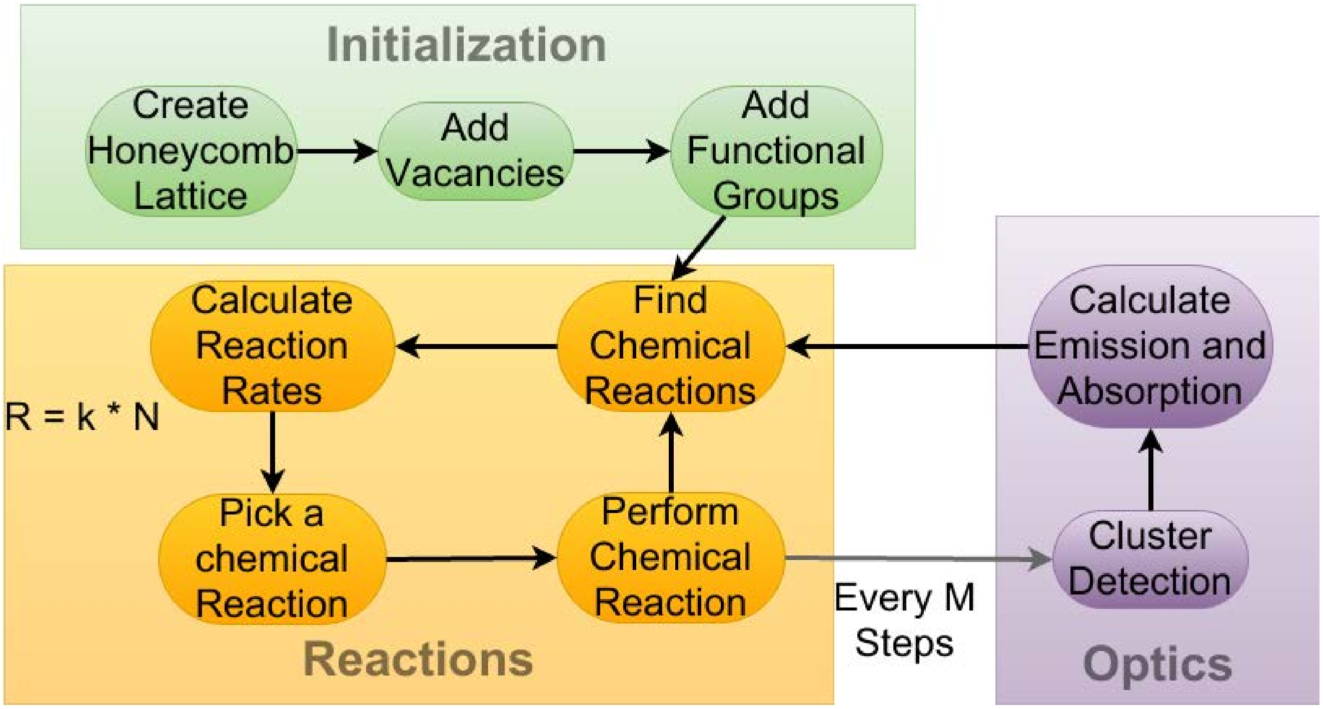

Supplementary Figure 2. Flow diagram of Monte Carlo Simulation. Simulation proceeds through initialization which creates a graphene oxide lattice, and then cycles through photoreduction reactions. Each reaction is weighted based upon the number, N, ways that the reaction can proceed (reactants) and by the rate constant for that reaction. Every M steps, the connectivity of the lattice is analyzed and clusters are identified. M is chosen to produce a sampling rate similar to the experimental sampling rate.

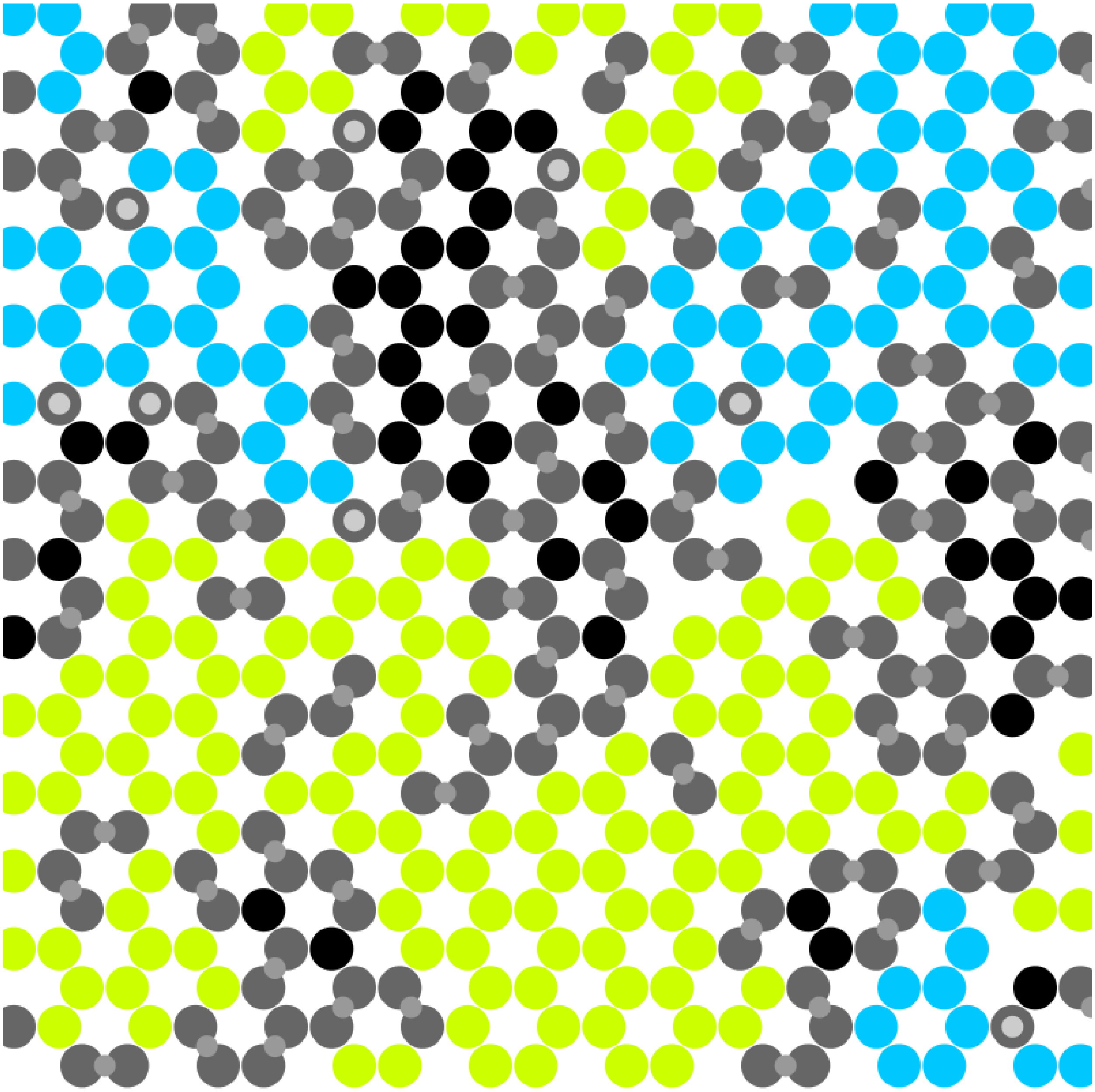

Supplementary Figure 3. Depiction of a single step in the Monte Carlo chemical reaction simulation The same parameterization was used as in figure 2a and 2b. The simulation contains a graphene sheet with most carbon sites filled in. The large circles in the honeycomb lattice represent carbon atoms and the smaller circles represent either OH groups or O atoms depending on their location. OH groups adhere above the atom, and epoxide (COC) forms above a bond. In this image, the black and grey carbons atoms are neither absorbing nor emitting and the colored carbon atoms are absorbing and emitting. Every grey carbon atom is considered non absorbing/emitting because it is in an sp^3 configuration with a surface oxide (either OH or O) attached to it. The yellow and blue clusters are separated by a wall of adsorbates. The black carbon atoms belong to sp^2 clusters that are too small to emit or absorb. There is also a third option (not depicted) where a cluster becomes too large to emit, but can still absorb.

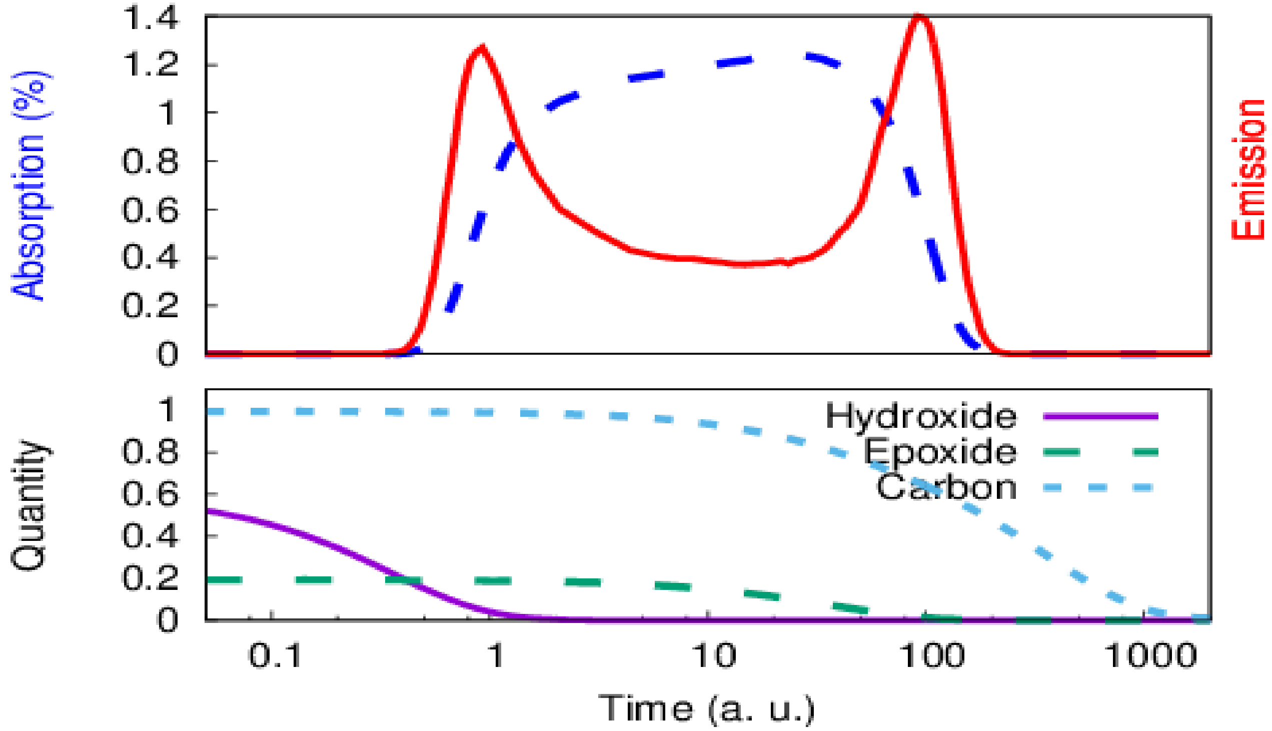

Supplementary Figure 4. Emission and absorption trajectories versus remaining reactants. The parameterization is the same as in figure 2a and 2b. The y axis of the second panel is a ratio between the number of that chemical species, and the initial number of carbon lattice sites.

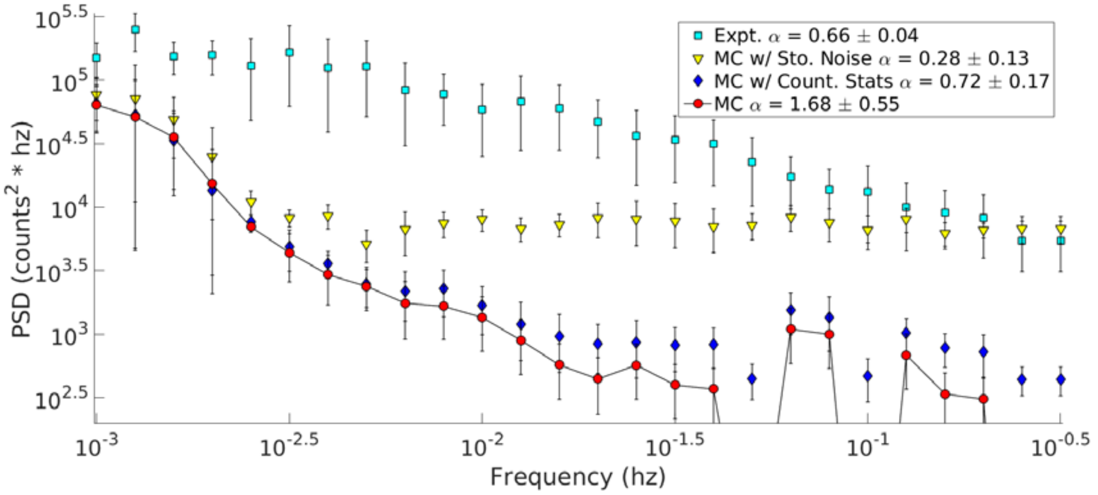

Supplementary Figure 5. Monte Carlo power spectrum altered by introducing Poisson noise. The parameterization of the MC matches figures 2c, 2d, 3, and 4 (The local structure model). The figure compares power spectral densities of the experiment, the MC simulation, the MC with counting statistics, and the MC with stochastic noise using a 500 sec window of the trajectory when blinking was strongest. Error bars are s.e.m. In each case, power law exponents are indicated in the legend. The generation of the trajectories with Poisson noise is discussed in Supplementary Note 3. For the raw MC PSD, a line has

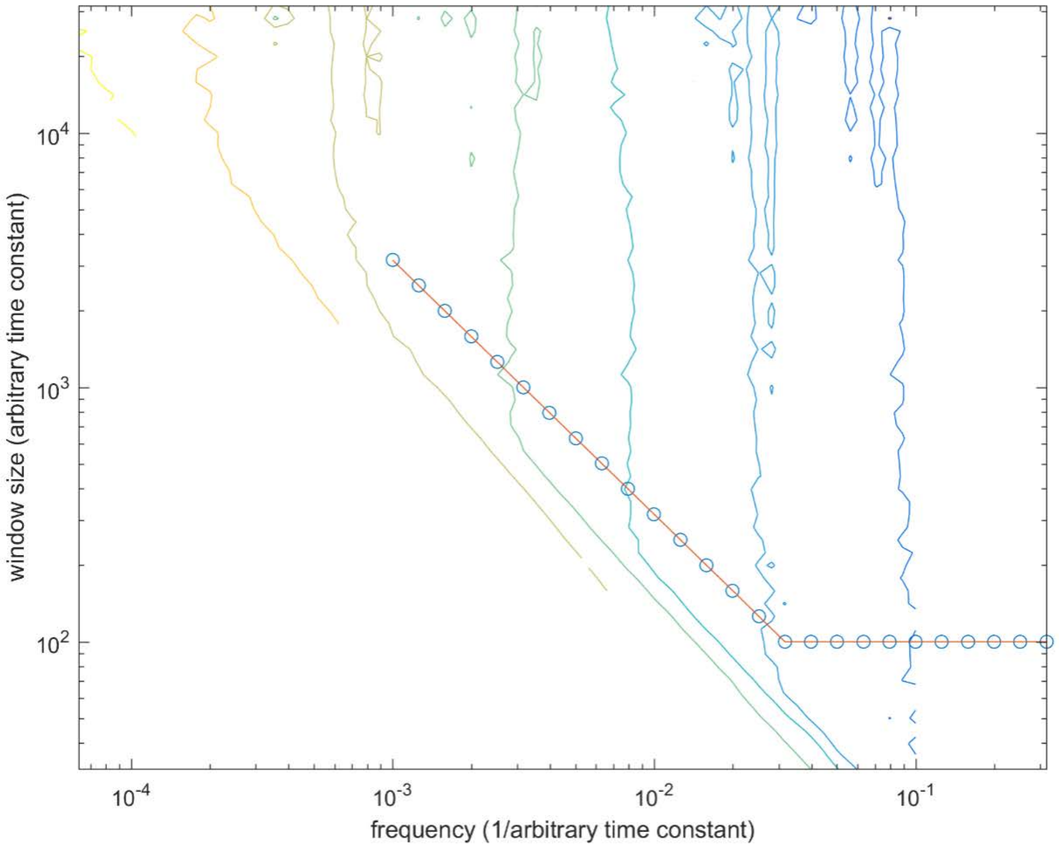

Supplementary Figure 6. Contour plot of the dependence of periodogram estimator on the choice of window size and frequency. The area showing near vertical contours of estimator values are the non-bias regions. The blue round dots connected by red line are examples of the choice of frequency and window size combination for PSD estimation. The method used to calculate PSD is given in Supplementary Methods 3.

Supplementary Tables

	$C_{24}H_{12}$	$C_{54}H_{18}$	$C_{96}H_{24}$	$C_{150}H_{30}$	$C_{216}H_{36}$
	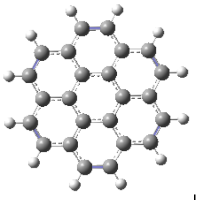	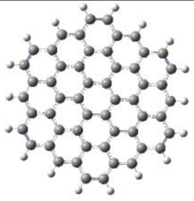	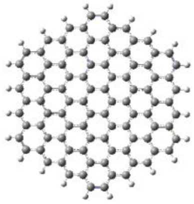	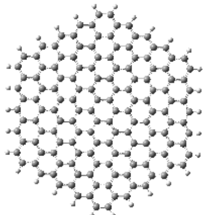	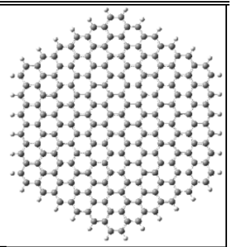
Diameter (nm)	0.74	1.23	1.73	2.22	2.71

Clar Number	3	7	12	19	27
Emission Energy(eV)	4.16	2.90	2.18	1.69	1.32
Radiation Lifetime	2.14 ns	2.95 ns	4.22 ns	6.31 ns	10.2 ns
Internal Conversion Lifetime	1.00 ns	422 ps	200 ps	80.0 ps	9.44 ps
Quantum Yield (%)	31.8	12.5	4.52	1.25	0.0925
Emission Intensity (a. u.)	7.63	6.75	4.34	1.88	0.20

Supplementary Table 1. Comparison of 5 carbon nanoclusters from 0.74 to 2.71 nanometers in diameter. The nanoclusters completely cover the visible range and have emission energies from infrared to ultraviolet. Although our TDDFT results are for absorption, the relative change in the electronic structure of the excited state is quite small. Consequently, the electronic transition energies for absorption and emission can be approximately regarded as the same. The calculation of radiative and internal conversion rates are given in Supplementary Methods 1 and 2 respectively.

Supplementary Notes

Supplementary Note 1: Photolysis and Rate Constants

Photolytic reactions follow selection rules, and rates of selected processes scale with temperature like thermally-activated Arrhenius rates. [1]

$$k = \mathrm{A}e^{-E_a/k_b\mathrm{T}} \quad \textbf{(1)}$$

E_a is an activation or barrier energy, k_b is the Boltzman constant, and A is a reaction dependent prefactor which varies from 10^{12} sec^{-1} to 10^{15} sec^{-1} for unary reactions or 10^{6} sec^{-1} to 10^{8} sec^{-1} for binary reactions. [1] The intensity of the laser used in the photoreduction experiments was only capable of raising the temperature by 1-2 °C. [2] The main features of photoreduction show up on timescales of 0.1s to 1000s. If we consider the maximum range of A, 10^{6} sec^{-1} to 10^{15} sec^{-1}, then the activation energies must lie within $0.35eV \leq E_a \leq 1.09eV$.

In this regard, theoretical activation energies for hydroxide desorption (0.54eV) [3], hydroxide dissociation into epoxide and water (0.5eV) [4], epoxide abstraction as O_2 (1.0eV) [4] [5], and oxygen (0.98eV) [6] or hydroxide (0.32eV) [3] hopping agree well with this energy range. However, the mechanism of lattice disintegration remains elusive because carbon sublimation from edges has activation energies of 4.5eV and 7.5eV for armchair and zigzag edges respectively [7]. A process for dissociation of carbon with more than one carbon neighbor is necessary for lattice disintegration. It has been demonstrated that the photo-Fenton [8] process can create holes in rGO. [9] A possible mechanism was proposed where adsorbed hydroxide groups migrate to edges and they assist in carbon abstraction through the formation of carboxyl (COOH) groups.

[10] Energy barriers for dissociation of carboxyl groups were calculated to be 0.57eV and 2.26eV for armchair and zigzag edges respectively. However, the energy for carboxyl dissociation on zigzag edges is still too high to be reachable. Additionally, another mechanism is necessary for conversion of edge carbon atoms which have 2 C-C bonds to single C-C bond carboxyl groups. Strain due to sp^3 bonding has been shown to cause wrinkling in GO. [9] Wrinkled graphene is more susceptible to an oxidative cutting reaction which has activation energies less than 1eV [11].

Even if we had energy barriers for all of the relevant reactions in the system, those energy barriers would still refer to ideal cases which differ greatly from the local structure in graphene oxide. For instance, the hydroxide hopping barrier was calculated for a single hydroxide molecule on an infinite graphene lattice with no defects, other functional groups, or vacancies. Low-barrier diffusion reactions can lead a molecule to a new lattice site with lower desorption barriers, and so the most relevant activation energies are for the lowest energy desorption accessible pathways. We propose that functional group abstraction happens primarily through groups hopping to edges or vacancies/defects and then desorbing. For these reasons, we treat the rate constants phenomenologically. We restrict the rate constants to within 4 orders of magnitude of each other and we order them based upon their idealized activation energies locking in well-known differences.

Supplementary Note 2: Chemical Reactions used in Monte Carlo

$$C_nOH \rightarrow C_n + OH^-, \qquad (\text{Rate} = k_{OH}) \qquad \textbf{(2)}$$

$$C_nO \rightarrow C_{n-1} + CO, \qquad (\text{Rate} = k_O) \qquad \textbf{(3)}$$

$$C_n \rightarrow C_{n-1} + C, \qquad (\text{Rate} = k_{C1}, k_{C3}, k_{Czig}, \text{ or } k_{Carm}) \qquad \textbf{(4)}$$

k_{C1} (the least stable) was applied to carbon atoms with a single nearest neighbor carbon, k_{C3} (the most stable) was applied to carbon with three nearest neighbor carbon atoms, k_{Czig} was applied to carbon atoms with 2 nearest neighbors and 4 next-nearest neighbors, and k_{Carm} was applied to carbon atoms with 2 nearest neighbors and less than 4 next-nearest neighbors.

Based upon the timescales present in the experimental data, the rate constants of the fastest removal process **(**Supplementary Equation 2**)** and the fastest process capable of destroying the carbon lattice must be within 4 orders of magnitude of each other. For instance even if k_{C3} = 0, then the other carbon removal processes are sufficient to disintegrate a lattice. However if k_{C3} = $k_{Czigzag}$ = $k_{Carmchair}$ = 0, then the remaining process, k_{C1}, is insufficient to disintegrate the lattice on its own. In the simulation a phenomenological estimation of rate constants was used, below a description of the connection between the rate constants and activation barriers is provided.

Figure 2a, 2b, and the yellow dashed-dotted line in figure 2d in the main text used the following rate constants: k_{OH} = 1000, k_O = 10, k_{C1}= k_{C3}= $k_{Czigzag}$ = $k_{Carmchair}$=1

The blue dotted line in figure 2d used the following rate constants: k_{OH} = 1000, k_O = 10, k_{C1}= 1, $k_{Czigzag}$ = $k_{Carmchair}$= 0.1, k_{C3}= 0

Figure 2c, the red dashed in figure 2d, and the data presented in figures 3 and 4 used the following rate constants: k_{OH} = 1000, k_O = 10, k_{C1}= 10, $k_{Carmchair}$=1, $k_{Czigzag}$ = 0.1, k_{C3}= 0

Supplementary Note 3: Monte Carlo with counting statistics and reversible processes

In order to account for both experimental counting statistics (shot noise) and the likely existence of reversible reactions, we consider the impact of introducing Poisson noise to the Monte Carlo results. In the former case, Poisson noise just models experimental shot noise. In the latter case, Poisson noise can be used to model the effects of chemical reversibility in equations (1-3) of the main text. Specifically, when reverse reaction timescales are comparable to the experimental integration time, variations in the number of active (emissive) domains will cause fluctuations to the overall integrated emission intensity. This will then manifest itself as apparent noise in the data. Hence, the inclusion of additional stochastic noise intensity variations to the Monte Carlo simulation approximates the tangible effects of chemical reversibility.

The following are implicit assumptions and limitations of the approximation that should be noted: (a) First, if reversible reaction rates are significantly faster than the experimental binning time (second timescale), intensity fluctuations stemming from these reversible reactions will be averaged out. (b) The stochastic noise approximation is based on many emitters with equal photoluminescence intensity, and it assumes that each switching event is a single emitter event. Therefore, the intensity distribution can deviate from a Poisson distribution. (c) Next, if reversible reaction rates are significantly slower than the experimental binning time, intensity fluctuations will exhibit time-correlations, which are not captured by Poisson noise. (d) The use of Poisson noise to represent the tangible effects of reversible reactions therefore implicitly assumes that reverse reaction rates exhibit timescales comparable to the experimental binning time. In the absence of detailed activation energies and a full accounting of *all* possible reversible reactions, beyond those associated with equations (1-3), an accurate estimation of relevant reverse reaction rates cannot be established. Thus, at present, a fully rigorous accounting of reversibility is beyond the scope of this study.

In practice, to include counting statistics, for each data point in the Monte Carlo trajectory, the number of photons that would have been counted in a detector is randomly generated by choosing a value from a Poisson distribution. The mean of the Poisson distribution is set to the number of counts (max ~3000) seen in the original MC trajectory. To include stochastic noise, associated with reversible reactions, the same methodology is applied. The number of active clusters is generated randomly by choosing a value from a Poisson distribution. The mean of the Poisson distribution in this case is given by the number of Monte Carlo-estimated emissive clusters (max ~300). Then the number of clusters is multiplied by the average counts/cluster to obtain a new estimate for the total number of counts observed. Fluctuations in cluster number thus more significantly impacts the Monte Carlo trajectories than variations due to counting statistics.

Supplementary Figure 5 shows the impact of adding Poisson noise to Monte Carlo trajectories. Namely, adding counting statistics to the Monte Carlo trajectory reduces its power law exponent to $\alpha = 0.7$. When only noise from reversible processes is considered, the power law exponent becomes even smaller $\alpha = 0.3$. It is therefore evident that improved modeling of reversible reactions will lead to better agreement between experiment and theory.

Supplementary Methods

Supplementary Methods 1 Life time vs. oscillator strength

The rate constant of spontaneous radiation transition is given by

$$A_{ng} = \frac{2e^2(\omega_{ng})^2}{4\pi\varepsilon_0 mc^3} f_{ng} \quad \textbf{(5)}$$

Or its corresponding life time is given by

$$\tau_{ng} = \frac{1}{A_{ng}} = 2.30508\times10^{-8} \frac{1}{f_{ng}(\omega_{ng}[\text{eV}])^2}\,. \quad \textbf{(6)}$$

where

f_{ng} The oscillator strength of the transition between the ground state g to the excited state *n*.

ω_{ng} Its corresponding transition frequency (s^{-1})

e: $1.602*10^{-19}$ C

ε_0 Permittivity of vacuum ($8.85418781762 * 10^{-12}$ F/m)

c Speed of light (299792458 m/s)

Supplementary Methods 2 Calculation of Internal Conversion Rate

The rate constant of the internal conversion is given, under single promoting mode approximation, by

$$W_{n\to g} = \left|\frac{R_{ng}^p}{\hbar}\right|^2 \frac{\omega_p}{2\hbar} \mathrm{Re}\int_0^\infty dt\, \exp[it(-\omega_{ng} + \omega_p) - \gamma t] G_{ng}(t) \quad \textbf{(7)}$$

where $R_{ng}^{p} = -\hbar^{2}\left\langle \Phi_g \left| \frac{\partial}{\partial Q_p} \right| \Phi_n \right\rangle$ is an electronic coupling matrix element due to the break-down of the Born-Oppenheimer approximation, Φ_g (Φ_n) is the electronic ground (excited) state wave function, $\hbar\omega_p$ and Q_p are the vibrational energy of the promoting mode and its normal coordinate, respectively, $\hbar\omega_{ng}$ is the electronic transition energy, and γ is a convergence factor. The term $G_{ng}(t)$ in Eq. (C-1) is given, $\hbar\omega_\ell >> k_B T$, by

$$G_{ng}(t) = \exp[-\sum_{\ell} S_\ell (1 - e^{it\omega_\ell})] \qquad \textbf{(8)}$$

Expansion of Eq. **(S4)** leads to

$$G_{ng}(t) = \exp[-\sum_{\ell} S_\ell] \sum_{n=0}^{\infty} \frac{(S_\ell)^n e^{itn\omega_\ell}}{n!} \sum_{m=0}^{\infty} \frac{(S_s)^n e^{itn\omega_s}}{m!} \cdots \qquad \textbf{(9)}$$

Substituting Eq. **(S5)** into Eq. **(S3)** yields

$$W_{n\to g} = | R_{ng}^{p} / \hbar |^2 \frac{\omega_p}{2\hbar} \exp[-\sum_{\ell} S_\ell] \sum_{n=0}^{\infty} \sum_{m=0}^{\infty} \cdots \frac{(S_\ell)^n}{n!} \frac{(S_s)^m}{m!} \vdots$$

$$\times \mathrm{Re} \int_0^{\infty} dt \exp[it(-\omega_{ng} + \omega_p + n\omega_\ell + m\omega_s + \cdots) - \gamma t] \qquad \textbf{(10)}$$

Under two-accepting-mode approximation, Eq. **(S6)** becomes

$$W_{n\to g} = | R_{ng}^{p} / \hbar |^2 \frac{\omega_p}{2\hbar} \exp[-(S_1 + S_2)] \sum_{n=0}^{\infty} \sum_{m=0}^{\infty} \frac{(S_1)^n}{n!} \frac{(S_2)^m}{m!}$$

$$\times \frac{\gamma}{(\omega_{ng} - \omega_p - n\omega_1 - m\omega_2)^2 + \gamma^2} \qquad \textbf{(11)}$$

If we assume $| R_{ng}^{p} / \hbar |^2 \frac{\omega_p}{2\hbar} \exp[-(S_1 + S_2)]$ does not depend on the size of graphene clusters, Eq. **(S7)** leads to

$$W_{n\to g} = A \sum_{n=0}^{\infty} \sum_{m=0}^{\infty} \frac{(S_1)^n}{n!} \frac{(S_2)^m}{m!} \frac{\gamma}{(\omega_{ng} - \omega_p - n\omega_1 - m\omega_2)^2 + \gamma^2} \qquad \textbf{(12)}$$

where $A = | R_{ng}^{p} / \hbar |^2 \frac{\omega_p}{2\hbar} \exp[-(S_1 + S_2)]$.

Supplementary Methods 3: Calculation of Power Spectral Density

The power spectral density (PSD) of the trajectory is calculated based on Welch's method.[12] First, the trajectory of total length $N$is divided into overlapping segments with length of M. The overlapping rates of the segments are chosen to be 50%, which means there are $K = N\ mod\ (M/2) + 1$ segments. Then the modified periodogram, as defined in **(S13)** is calculated for each of the segments.

$$\hat{S}_M(f) = \frac{\Delta t_0}{M} \left(\sum_{n=1}^{M} w_n x_{mM-n+1} \exp\{i2\pi f \Delta t_0 (n-1)\} \right)^2 \quad \textbf{(13)}$$

Here, Δt_0 is the unit time difference between discrete temporal signals, w_n is the window function that convolves with the signal trajectory, for which we used Hamming window in our calculation. Then the periodograms are averaged to reduce the variance in the periodogram estimator.

$$\hat{S}_{MAP}(f) = \frac{1}{K} \sum_{m=1}^{K} \hat{S}_m(f) \quad \textbf{(14)}$$

Before the estimation of the trajectory PSD, the dependence of window size and frequency of the periodogram estimator is evaluated to find the no-bias region of the estimator, as shown in **Figure S6**. A small value for the window size of a given frequency is chosen in the non-bias region to minimize the variance.

Since the trajectory of blinking shows long range self-correlation, we utilize an extended version of central limit theorem in estimation of the variance of the periodogram estimator, in which

$$\hat{\kappa}_{MAP}(f) = \frac{1}{K} \left(\hat{\kappa}_0(f) + 2 \sum_{m=1}^{m_c} \left(1 - \frac{m}{K}\right) \hat{\kappa}_m(f) \right) \quad \textbf{(15)}$$

$$= \frac{1}{K-m} \sum_{n=1}^{K-m} \hat{S}_n(f) * \hat{S}_{n+m}(f) \quad \textbf{(16)}$$

are defined. Variance of the periodogram estimator is determined by the maximum value of $\hat{\kappa}_{MAP}(f)$ when m_c goes from 1 to K.

Supplementary References

[1] Turro, N. J. Modern Molecular Photochemistry. *University Science Books:* Sausalito (1991)

[2] Sokolov, D. A., Morozov, Y. V., McDonald, M. P., Vietmeyer, F., Hodak, J. H., & Kuno, M. Direct Observation of Single Layer Graphene Oxide Reduction through Spatially Resolved, Single Sheet Absorption/Emission Microscopy. *Nano Lett.*, **14,** 3172-3179 (2014).

[3] Ghaderi, N. & Peressi, M. First-Principle Study of Hydroxyl Functional Groups on Pristine, Defected Graphene, and Graphene Epoxide. *Jour. Phys. Chem. C,* **114,** 21625-21630 (2010)

[4] Zhou, S. & Bongiorno, A. Origin of the Chemical and Kinetic Stability of Graphene Oxide. *Sci. Rep.,* **3,** 2484 (2013)

[5] Sljivancanin, Z., Milosevic, A. S., Popovic, Z. S. & Vukajlovic, F. R. Binding of Atomic Oxygen on Graphene from Small Epoxy Clusters to a Fully Oxidized Surface. *Carbon,* **54,** 482-488 (2013)

[6] Radovic, L. R., Suarez, A., Vallejos-Burgos, F., & Sofo, J. O., Oxygen Migration on the Graphene Surface. 2. Thermochemistry of basal-plane diffusion (hopping). *Pergamon-Elsevier Sci. Ltd.* **49,** 4226-4238 (2011).

[7] Huang, J. Y., Ding, F., Yakobson, B. I., Lu, P., Qi L., & Li, J. In situ Observation of Graphene Sublimation and Multi-layer Edge Reconstructions. *PNAS* **106,** 10103-10108 (2009)

[8] Guenes, F., Han, G. H., Shin, H. et al. UV-light-assisted Oxidative sp3 Hybridization of Graphene. *Nano,* **6**, 409-418 (2011).

[9] Radich, G. J. & Kamat, P. V. Making Graphene Holey. Gold-Nanoparticle-Mediated Hydroxyl Radical Attack on Reduced Graphene Oxide. *ACS Nano,* **7,** 5546-5557 (2013)

[10] Bai, H., Jiang, W., Kotchey, G. P., et al. Insight into the Mechanism of Graphene Oxide Degradation via the Photo-Fenton Reaction. *Jour. Phys. Chem. C*, **118,** 10519-10529 (2014)

[11] Tan, X. & Zapol, P. Regioselective Oxidation of Strained Graphene for Controllable Synthesis of Nanoribbons. *Jour. Phys. Chem. C,* **117,** 19160-19166 (2013)

[12] Frantsuzov, P. A., Volkan-Kacso, S., & Janko, B., Universality of the Fluorescence Intermittency in Nanoscale Systems: Experiment and Theory. *Nano Lett.*, **13,** 402-408 (2013).
